# The Role of Oxidative Stress in the Pathogenesis of Vitiligo: A Culprit for Melanocyte Death

**DOI:** 10.1155/2022/8498472

**Published:** 2022-01-22

**Authors:** Yijie Xuan, Yiwen Yang, Leihong Xiang, Chengfeng Zhang

**Affiliations:** Department of Dermatology, Huashan Hospital, Fudan University, 12 Wulumuqi Zhong Road, Shanghai 200040, China

## Abstract

Vitiligo is a common chronic acquired pigmentation disorder characterized by loss of pigmentation. Among various hypotheses proposed for the pathogenesis of vitiligo, oxidative stress-induced immune response that ultimately leads to melanocyte death remains most widely accepted. Oxidative stress which causes elevated levels of reactive oxygen species (ROS) can lead to dysfunction of molecules and organelles, triggering further immune response, and ultimately melanocyte death. In recent years, a variety of cell death modes have been studied, including apoptosis, autophagy and autophagic cell death, ferroptosis, and other novel modes of death, which will be discussed in this review in detail. Oxidative stress is also strongly linked to these modes of death. Under oxidative stress, ROS could induce autophagy by activating the Nrf2 antioxidant pathway of melanocytes. However, persistent stimulation of ROS might eventually lead to excessive activation of Nrf2 antioxidant pathway, which in turn will inactivate autophagy. Moreover, ferroptosis may be triggered by oxidative-related transcriptional production, including ARE, the positive feedback loop related to p62, and the reduced activity and expression of GPX4. Therefore, it is reasonable to infer that these modes of death are involved in the oxidative stress response, and that oxidative stress also acts as an initiator for various modes of death through some complex mechanisms. In this study, we aim to summarize the role of oxidative stress in vitiligo and discuss the corresponding mechanisms of interaction between various modes of cell death and oxidative stress. These findings may provide new ideas for exploring the pathogenesis and potential therapeutic targets of vitiligo.

## 1. Introduction

Vitiligo is an acquired pigmentation disorder of unknown origin and the most frequent cause of depigmentation, causing an estimated prevalence between 0.5 and 2% worldwide [1]. Although vitiligo does not affect the survival of patients, it can bring social pressure and cause psychological disorders, which greatly interfere with the life quality of patients. Over the past decades, numerous studies have attempted to illustrate the pathogenesis behind vitiligo, including the degenerative theory, genetic factors, oxidative stress, autoimmune hypothesis, and cell detachment mechanisms. Recent studies further demonstrated that innate immunity is likely to work as the bridge between oxidative stress and autoimmunity [2]. Several researchers have evaluated the influence of alopecia areata and/or autoimmune thyroid disease on vitiligo by using the biomarkers [3, 4]. It seems that vitiligo patients do have an increased risk of developing other autoimmune diseases. Surprisingly, even fibroblast has proved to play an essential role in the pathogenesis of vitiligo [5]. However, these theories are insufficient to explain the pathogenesis of vitiligo individually, and the contribution of each hypothesis is still under debate [6–8]. The oxidative stress hypothesis showed that reactive oxygen species (ROS) are induced by multiple factors and impair antioxidant defenses, breaking the melanocyte redox homeostasis, which may contribute to the onset of vitiligo. Excessive production of ROS leads to the imbalance of the antioxidation system in melanocytes, and eventually, cell damage. One study provided evidence that the serum oxidative stress indicator (total antioxidant capacity (TAC), malondialdehyde (MDA), and 8-hydroxy-2′-deoxyguanosine (8-OHdG)) can indicate the activity and severity in patients with NSV [9]. Despite different theories of pathogenesis, damage of melanocytes is the coincident outcome in vitiligo.

Over the decades, studies have revealed that multiple types of melanocyte death including apoptosis, necrosis, autophagy, necroptosis, pyroptosis, ferroptosis, anoikis, and phagoptosis might be involved in vitiligo [10]. In this review, we also summarized the role of oxidative stress in several death modes of vitiligo melanocytes. This review also provides new insight for the investigation of the potentially broad application of the cellular cross-talk in vitiligo.

## 2. ROS and Antioxidant Defense System in Vitiligo

Although a large number of theories have been proposed in vitiligo, there is no consensus on the exact etiology of how melanocytes are destroyed and eventually dead. Oxidative stress plays a central role in initiating the onset of vitiligo with melanocytes damage. It is oxidative stress that triggers the imbalance of redox homeostasis, manifested by excessive formation and inadequate scavenging of reactive oxygen species (ROS). ROS is stimulated by endogenous and exogenous stimuli and finally causes melanocyte death. During the process of melanogenesis, excessive ROS is produced to form a prooxidative atmosphere, rendering the melanocytes easily attacked by oxidative stress [11].

A high concentration of ROS is implicated in murdering melanocytes in all aspects, including undermining their DNA, lipid, protein, and metabolites structurally and functionally [12]. Furthermore, ROS-induced oxidative stress widely instigates aberrant organelle functions, derails metabolism pathways, and compromises defensive mechanisms against the onslaught of oxidative agents. To comprehend the mechanisms responsible for oxidative stress and its association with melanocyte obliteration observed in vitiligo, we will illustrate these aspects in detail.

### 2.1. Sources of ROS Overproduction in Vitiligo

The generation of highly enriched ROS to which melanocytes are subject can be attributed to two aspects, excessive formation, and inadequate scavenging. Endogenous and exogenous stimuli contribute to the excessive formation of ROS. From the perspective of exogenous stressor, overproduction of ROS is partly triggered by various factors, including exposure to the environment (e.g., ultraviolet irradiation, PM2.5, cytotoxic chemicals, and trauma), other diseases (malignancies, major infection, neural disorders, and calcium imbalance), and medication application (e.g., certain drugs, hormones, and vaccination) [2, 13, 14]. The production of ROS is an instant reaction after exposure to stressors. On the other hand, ROS can be attributed to a series of inner stimuli: (a) cellular metabolic processes, which are genetically determined, such as melanogenesis that demand more energy during this process; (b) abnormal energy metabolism in mitochondria, which eventually leads to cellular proliferation/differentiation/apoptosis [15, 16]. In the process of melanogenesis, ROS generation ensues from the conversion of dopa to dopaquinone and then to dopachrome, rendering melanocytes more susceptible to oxidative damage [17]. Besides, melanogenesis is an energy-consuming procedure, demanding large amounts of adenosine triphosphate (ATP). The biogenesis of ATP itself, accompanied by ROS production in mitochondria, forms a prooxidative atmosphere in epidermis. Redox imbalance of membrane lipids may lead to a compromised function and altered structure, which could affect intracellular transduction mediated by membrane receptors, electron transport, and mitochondrial energy [18]. Collectively, these changes put melanocytes at the center of the accumulation of ROS.

Other than excessive formation, aberrant ROS removing mechanisms also account for superabundant ROS in the epidermis. Nature has evolved three antioxidative layers to eliminate ROS. The first barrier is some small-molecule antioxidants, such as uric acid, glutathione (GSH), and vitamins C and E, which offer the first line of defense to scavenge ROS/RNS directly and thus prevent or delay the initiation of various oxidative stresses. Damage-removing or repairing enzymes function as the last defense to regenerate biomolecules damaged from oxidative injury. Between them, antioxidant enzymes serve as an intermediate defense [19]. Many studies have demonstrated that there is an imbalance of the antioxidant system in patients with vitiligo. The level of ROS increases in the skin lesions of vitiligo, while the expression of antioxidant enzymes such as catalase (CAT), glutathione reductase (GPX), thioredoxin (Trx)/thioredoxin reductase (TrxR), and methionine sulfoxide reductase (Msr) A and B is downregulated [20, 21]. The activities of these enzymes are significantly reduced in the skin lesions of patients with vitiligo, which further aggravates the accumulation of ROS. It is worth mentioning that other pathways may participate in the protection of melanocytes from oxidative damage. Several experiments illustrated the destruction of nuclear factor E2-related factor 2-antioxidant response element/heme oxygenase-1 (Nrf2-ARE/HO-1) pathway in melanocytes and keratinocytes in vitiligo [22], which will be further described in the following chapters.

### 2.2. Consequences of Excessive ROS in Vitiligo

#### 2.2.1. The Oxidative Damage of Biological Macromolecules

It has been demonstrated that a high concentration of ROS triggered oxidative stress in the epidermis of vitiligo patients. Concerning the influences of oxidative stress on the onset of vitiligo, a reasonable hypothesis points out that oxidative stress could damage the biological macromolecules in melanocytes of the epidermis and then cause functional loss of melanocytes. As extremely active radicals, ROS potently damages most biological macromolecules and leads to the formation of DNA-protein cross-links, DNA breaks, lipid peroxidation, protein oxidation/fragmentation, and enzyme activation/inactivation [23]. A series of researches incorporated advanced oxidation protein products (AOPPs), advanced glycation end-products (AGEs), propanediol, and 8-hydroxy-2′-deoxyguanosine (8-OHdG, a major form of oxidative DNA damage) as markers of progressive vitiligo [24, 25]. The diffusibility of by-products of lipid peroxidation allows for the formation of adducts with cellular proteins or DNA, which are known as advanced lipoxidation end products (ALEs), thereby amplifying oxidative damage [26]. It is worth noting that chronic oxidative stress stimulates perpetuating DNA damage reactions, which need to be eliminated by enhancing base excision repair, such as upregulating apurinic/apyrimidinic endonuclease 1 (APE1). Studies have revealed that the APE1 polymorphism (Asp148Glu) aggravates the oxidative stress of human melanocytes and increases serum 8-OHdG levels in an allelic dose-dependent manner, thereby increasing the risk of vitiligo [27]. However, the autoantigen of vitiligo is mainly derived from melanosomal proteins rather than damaged DNA, meaning that the autoantigen of damaged DNA probably plays an assistant role during the trigger of autoimmunity. After that, melanocytes can be manipulated to oxidative stress-related aging and apoptosis, with irreversible cell proliferation block [6]. In this context, senescent melanocyte exhibits a special “senescence-associated secretory phenotype (SASP)” and expresses a multitude of immune-related factors including interleukin- (IL-) 6, cyclooxygenase, extracellular matrix metalloproteinases (MMPs), and insulin-like growth factor-binding protein (IGFBP) [28–30]. We could presume that some of these secretions can remove the “senescent melanocytes” by accumulating immune cells, which may be a potential way to destroy melanocytes in a vicious cycle.

Massive accumulations of hydrogen peroxide (H_2_O_2_) in vitiligo skin have been illustrated by a host of *in vivo* and *in vitro* studies. Research has elucidated the influence of H_2_O_2_ on dihydropteridine reductase (DHPR), the last enzyme in the 6BH4-recycling process. The involvement of H_2_O_2_ can cause deactivation of DHPR, leading to disruption of the NADH-dependent enzyme active site and consequently causing the synthetic and cyclic defection of biopterin, which finally destroys melanocytes [31]. From another perspective, H_2_O_2_ can affect pigmentation via epidermal proopiomelanocortin (POMC) peptide (a possible target for oxidation by this reactive oxygen species) redox homeostasis [32]. Deactivating and deregulating acetylcholinesterase (AchE) due to H_2_O_2_-mediated oxidation further maintains epidermal oxidative stress. Therefore, H2O2 is a key component of ROS and plays an essential role in damaging biological macromolecular in vitiligo by a variety of mechanisms.

Oxidative damage of macromolecular substances can be considered as a bridge between oxidative stress and subsequent immune response in vitiligo. In summary, during the process of vitiligo, enhanced ROS is produced, which mediates biological macromolecules alteration to maintain persistent damage melanocytes and probably triggers the production of autoantigen.

#### 2.2.2. Stressed Organelles

It is widely concerned that mitochondria are not only the place where ROS is produced but also the victim of the damage of oxidative stress. Accordingly, mitochondrial impairment is bound to affect melanocyte survival.

Abnormal lipid composition of mitochondria and impaired respiratory chain integrity is also induced by oxidative stress, which is the biochemical basis of intracellular ROS production after mitochondrial damage and mild stress [18]. In association with this process, the shell of the electron transport chain on the lipid could be modified. Such potential modulation of the energetic pathways by small molecules can interfere with mitochondrial lipid membrane assessment. Therefore, the functional and energetic defects occurring at the mitochondrial level may be the initial step leading to the defective functional profile [33]. Due to oxidative stress-derived deficiency in assembly and intrinsic vulnerability to oxidative injury, mitochondrial complex, especially complex I, exhibits dysfunction and further induces more ROS production, thus, forming a vicious spiral to destroy melanocytes and to elevate the sensibility to proapoptotic stimuli [34]. Above all, more and more evidence has shown that mitochondria is indispensable to the death of melanocytes mediated by oxidative stress.

Endoplasmic reticulum (ER) is an extremely active organelle owning multiple functions. It is not only an important site where protein synthesis, folding, decoration, and transportation are accomplished but also in charge of cell homeostasis, development, stress sensation, and cell survival or death regulation [35]. Various stimuli produced by the external/internal environment or excessive protein will cause client protein to unfold, ultimately leading to the accumulation of protein misfolding or unfolding in the ER, which is called “ER stress.” Among all kinds of interference, oxidative stress plays an important role in triggering ER stress. The production of ROS increases the risk of protein unfolding and misfolding in ER. It is concerned that the accumulation of unfolded protein in ER and/or the imbalance of cellular Ca^2+^ homeostasis activates unfolded protein action (UPR) [36]. UPR comprises three parallel signaling branches: inositol-requiring enzyme 1*α*- (IRE1-) X-box-binding protein 1 pathway, activating transcription factor 6 (ATF6) pathway, and PRKR-like ER kinase- (PERK-) eukaryotic translation initiation factor 2*α* pathway. All the major arms of UPR have a central role in immune regulation. Continuously activated by oxidative stress-induced ER stress, the three branches might work independently or in concert to elicit apoptosis.

To combat ER stress the melanocytes activate UPR which may alleviate ER stress through global translation attenuation, induction of chaperones, degradation of misfolded proteins by ER-associated degradation (ERAD), and apoptosis [37]. However, excessive and prolonged UPR may contribute to cell death. The specific mechanism is probably that TRAF2-mediated activation of NF-*κ*B and JNK1/2 helps to safeguard cells from apoptosis by attenuating ROS production [38]. Thus, oxidative stress may induce endoplasmic reticulum stress and accumulation of UPR, eventually activate the apoptotic pathway.

The effect of oxidative stress may also influence other macromolecules and prompt the progress of autoimmunity. Under certain pathophysiological conditions, several ER chaperones (such as HSP70i) may act as the damage-related molecular pattern (DAMPS) and attract the innate immune system to target “abnormal” cells for phagocytosis, leading to subsequent activation of adaptive immunity [39, 40]. Importantly, simultaneous inhibition of endoplasmic reticulum stress and HSP70i release can protect melanocytes from H_2_O_2_-induced oxidative damage [41], indicating that the endoplasmic reticulum stress pathway and molecular chaperones may be therapeutic targets for vitiligo.

### 2.3. The Role of Nrf2-ARE in Vitiligo

Nuclear erythroid 2-related factor 2 (Nrf2), a redox-sensitive transcription factor, is crucial in governing redox homeostasis in cells [42]. The activity of Nrf2 is strictly regulated by its cytoplasmic repressor Kelch-like ECH-associated protein 1 (Keap1). Keap1 is the substrate recognition subunit of Keap1-cullin3 (Keap1-cul3) E3 ubiquitin ligase. The ubiquitin ligase binds to Nrf2 to make it ubiquitinated, which is targeted for degradation by 26S proteasome. Normally, Nrf2 and Keap1 combine in the cytoplasm and are in a state of inhibition. When the cell is under oxidative stress, the Keap1-Nrf2 complex will break down, and then Nrf2 can be transported to the nucleus and bind to antioxidant response elements (AREs) of gene promoters. This initiates the transcription of more than 200 antioxidants and phase II detoxifying enzymes and other cytoprotective genes, including NAD(P)H: quinone oxidoreductase 1 (NQO1), HO-1, glutathiones-transferase (GST), catalase, superoxide dismutase (SOD), and glutamate cysteine ligase modified subunits [43–45]. Therefore, Keap1/Nrf2/ARE pathway serves as the main mechanism for cells to resist oxidative stress and participate in the maintenance of cellular homeostasis.

Several studies have shown that the Nrf2 pathway plays an important role in protecting melanocytes from oxidative stress damage [46]. The Nrf2-ARE signal transduction in melanocytes of vitiligo is disordered, and the activation of the antioxidant enzyme system is reduced. Based on genetic studies of the Chinese Han population, Nrf2 gene polymorphism was associated with susceptibility to vitiligo [47]. Previous studies showed that the transcription level of Nrf2 and downstream genes, like NQO-1, *γ*-GCLC, and GCLM, was upregulated in vitiligo [48]. Nrf2-ARE antioxidant pathway and its downstream antioxidant enzyme HO-1 are crucial for the response of melanocytes to oxidative damage induced by H_2_O_2_. Aspirin, simvastatin, and other drugs can improve the viability of melanocytes under H_2_O_2_-induced oxidative stress by activating Nrf2 [49, 50]. The reduced activity of Nrf2-ARE leads to the decrease of downstream antioxidant enzymes and the increase of oxidative stress damage, rendering melanocytes more vulnerable to oxidative stress damage, while overexpression of Nrf2 protects melanocytes against oxidative stress. The above findings validated the correlation between the Nrf2-ARE pathway and oxidative stress in vitiligo and its role in melanocyte destruction, opening the possibility of targeting the Nrf2-ARE pathway as a promising therapeutic strategy against melanocyte oxidative damage.

### 2.4. Keratinocyte Changes Triggered by Oxidative Stress

Keratinocytes are not only the main component of epidermis but also interact with melanocytes. A growing number of studies have shown that keratinocytes are mediators of oxidative stress damage, secreting cytokines to recruit autoreactive T cells and interfering with melanocyte signal transduction, leading to melanocyte destruction [51, 52]. Oxidative stress triggers the overexpression of miR-25 in keratinocytes and melanocytes, which leads to reduced antioxidant enzyme activity and impaired melanosome transport, thus, giving rise to redox imbalance [53]. Disruption of the paracrine network in the melanocyte microenvironment is exemplified by reduced WNT, POMC, SCF, increased production of multiple cytokines by epidermal keratinocytes, and increased production of DKK1 and HGF by dermal fibroblasts, further increasing the susceptibility of melanocytes to apoptosis and exacerbate the autoimmune response. Similarly, oxidative stress stimulated keratinocytes to release HMGB1 signal transduction by downregulating the activity of melanogenetic molecules (such as gp100) and activating apoptosis. Parenthetically, under oxidative stress, HMGB1 translocalization from the melanin nucleus to the cytoplasm can also induce apoptosis by inhibiting Nrf2 expression [54]. These changes demonstrate the role of oxidatively stressed keratinocytes in interfering with melanocyte signal transduction. During the process of vitiligo, above keratinocyte-related mechanisms may trigger or exacerbate melanocyte loss in a parallel or hierarchical manner.

## 3. Forms of Melanocyte Death Induced by Oxidative Stress in Vitiligo

### 3.1. Apoptosis

Apoptosis, as the best-characterized form of programmed cell death, can be triggered by both physiologic and pathologic stimuli, which initiate multiple signaling pathways regulated by activated caspases and also molecular systems such as Bcl-2/bax and Fas/Fas ligand. Among the caspase family members, caspase-3 is a key mediator of apoptosis in mammalian cells. Among the Bcl-2 family, Bcl-2 can protect cells from numbers of apoptotic stimuli, while Bax is an apoptosis agonist that acts via hetero-dimerization with Bcl-2. It was reported that a lower ratio of Bcl-2/Bax was detected in perilesional melanocytes as compared to the control melanocytes, which signifies that altered expression of Bcl-2 and Bax might render melanocytes from vitiligo patients susceptible to the induction of apoptosis [55].

It has been widely accepted that excessive oxidative stress can lead to apoptosis in melanocytes, mainly involved in the intrinsic apoptosis signaling pathways, also called the mitochondrial pathway. This process can initiate apoptosis under a diverse array of nonreceptor-mediated stimuli that produce intracellular signals which can act directly on targets within the cell. Mitochondrial dysfunction leads to loss of the mitochondrial transmembrane potential, open of calcium channel, and release of cytochrome (Cyt) C into the cytosol, which then initiates the formation of a complex, called apoptotic protease activating factor-1 (Apaf-1) thereby activating caspase-9, in turn activating caspase-3 and eventually triggers cell apoptosis, making it a mitochondrial-initiated event [56]. Extracellular/intracellular ROS manipulates the overexpression of transient receptor potential cation channel subfamily M member 2 (TRPM2) and mediates Ca^2+^ influx into the cytoplasm and then promotes mitochondrial-dependent apoptosis [57]. Moreover, Baicalein, an antioxidant that proved beneficial to patients with vitiligo, was found to protect melanocytes by reducing the release of Cyt C, the Bax/Bcl-2 ratio, and caspase-3 level in a concentration-dependent manner in vitro [58]. On the other hand, the extrinsic apoptosis signaling pathways that initiate apoptosis involve transmembrane receptor-mediated interactions. The involved apoptosis pathways like the Fas/FasL interaction mediated by CD8 + T cell, the TNF-*α*/TNFR pathway, the IFN-gamma signaling pathway, and the CXCL10/CXCR3B pathway have indicated the crucial role of autoimmunity in the pathogenesis of vitiligo [59–61].

### 3.2. Autophagy and Autophagic Cell Death

Autophagy, also known as “self-eating,” is a lysosomal-dependent degradation pathway widely found in eukaryotic cells. Under various extreme stress conditions, it can deliver cytosol and/or its specific content (long-lived proteins and unwanted organelles) to the lysosomes for degradation [62].

Autophagy can be upregulated in response to extracellular or intracellular stress and signals, such as hunger, hypoxia, oxidative stress, pathogen infection, protein aggregation, ER stress, and UPR accumulation [63]. Meanwhile, autophagy is involved in the elimination of toxic molecules produced by oxidative stress. It is generally believed that oxidatively modified proteins are more or less degraded by the proteasome system. Instead, there is evidence that moderately or heavily oxidized proteins are degraded by the endosomal/lysosomal system. This suggests that proteins modified by oxidative stress are degraded primarily through cooperative proteasome and autophagosome/lysosomal pathways. P62, also known as sequestosome 1/SQSTM1, is one of the most abundant substrates for selective autophagy and is a ubiquitously expressed cellular protein. Autophagy is impaired with the accumulation of p62. This leads to the formation of large aggregates, including p62 and ubiquitin. The functions of p62 include the activation of the TNF receptor-associated factor 6- (TRAF6-) NF-*κ*B pathway to determine cell survival or death by promoting the aggregation of caspase-8 and downstream effector caspase effector [64]. Dysfunction of autophagy in melanocytes may occur in vitiligo, which affects the expression of functional molecules and is related to the clinical type of vitiligo. The genetic basis of autophagy deficiency may also be involved in the pathogenesis of vitiligo. In addition, a study reported that keratinocytes and scarce residual melanocytes in vitiligo lesions had significantly more autophagy vacuolation than normal and nondisaffected skin, suggesting that autophagy may play a role in the pathogenesis of vitiligo [65]. Intriguingly, it has been revealed that the level of autophagic activity correlates with defects in mitochondrial metabolism and the senescent phenotype of vitiligo melanocytes and fibroblasts [66]. However, the exact role of this process in the disease remains unclear.

Antioxidation drugs, such as calcipotriol and madecassoside, could take effect by activating autophagy, which was verified by the increase of Beclin1 and LC3-II/LC3-I in melanocytes [67]. It has been observed that, compared to normal melanocytes treated with H_2_O_2_, the level of LC3-II and the ratio of LC3-II/LC3-I are relatively low in vitiligo melanocytes with significantly fewer cytoplasmic vesicles [21]. Therefore, it is reasonably speculated that vitiligo melanocytes may have autophagy defects, and oxidative stress plays a related role in this process.

Besides the growing evidence showing that autophagy can exert cytoprotective effects, a contribution of autophagy to a mode of cell death defined as autophagic cell death has been proposed [68]. Although autophagy often accompanies cell death following numerous toxic insults, the requirement of autophagic machinery for cell death execution, as established through specific genetic or chemical inhibition of the process, is highly contextual [69]. Autophagic cell death rather than cell death with autophagy is another condition. Many studies have shown that the IL-17 level is higher in the serum and lesions of vitiligo patients compared to healthy individuals, suggesting its possible role in the pathogenesis of vitiligo [70]. The elevated IL-17 in lesions might contribute to the more autophagic vacuoles in melanocytes, and the autophagic process might be involved in the death of melanocytes in vitiligo by the IL-17 mediated ROS autophagy-associated cell apoptosis. Therefore, the process of autophagy may be involved in the death of melanocytes in vitiligo, while the existence of autophagic cell death is still under debate.

### 3.3. Ferroptosis

Ferroptosis is a newly identified form of iron-dependent cell death, which is remarkably distinct from apoptosis, autophagy, pyroptosis, and necroptosis in morphological, biochemical, and genetic aspects. Because of its obvious iron dependence and the accumulation of ROS and lipid peroxides in cells, it is called ferroptosis [71].

It is now known that ferroptosis occurs through three major steps: reduced activity of the cysteine glutamate antiporter (System Xc^−^), inhibition of glutathione peroxidase 4 (GPX4), and iron-dependent generation of lipid peroxides. Eradicator of RAS- and ST-expressing cells (erastin) and Ras Selective Lethal 3 (RSL3) was the first ferroptosis inducing compounds. During their subsequent characterization, both erastin and RSL3 were noted to increase cellular reactive oxygen species (ROS) and elicit specific morphological responses like mitochondrial shrinkage in the absence of any other forms of cell death such as chromatin condensation, cytoplasmic swelling, or plasma membrane rupture. Also, a distinct set of small molecules consisting of deferoxamine (an iron chelator, DFO), Trolox (a vitamin E analog), ebselen (a glutathione peroxidase mimetic), and U0126 (a MEK inhibitor) protected cells from erastin and RSL3 induced cell death. Two novel small molecules, named ferrostatin-1 and liproxstatin-1, also inhibited the generation of lipid peroxides than restrained this form of cell death as radical trapping agents (RTAs) [71].

Interestingly, Nrf2 is tightly connected to the ferroptosis pathway. The Nrf2 transcription factor is not only a master regulator of the antioxidant response but also responsible for regulating iron metabolism (e.g., FTH, FTL, and SLC40A1) and preventing lipid peroxidation (e.g., SLC7A11, GPX4, and FSP1) by targeting its downstream genes [72]. Studies have shown that ferroptosis is closely related to the pathophysiological process of many diseases such as tumors, nervous system diseases, ischemia-reperfusion injury, kidney damage, and ischemic diseases. The target organs of these diseases share a common feature of abnormal accumulations of PUFA lipids or iron overload and lipid peroxidation under oxidative stress stimulation, which increases the susceptibility to ferroptosis. In recent years, studies revealed that ferroptosis may also be involved in the death of melanocytes in vitiligo [10]. In skin, melanocytes have a higher content of bioavailable iron than keratinocytes. Under external oxidative stimulation, the level of Fe^2+^ in melanocytes and the oxidation of unsaturated fatty acids increase at the same time, which greatly improves the susceptibility of melanocytes to ferroptosis. Therefore, despite the current lack of evidence, we can also speculate that ferroptosis is involved in the damage of melanocytes in vitiligo and plays a certain role in the pathogenesis of vitiligo.

### 3.4. Other Forms of Cell Death in Vitiligo Related to Oxidative Stress

Melanocyte loss is considered to be the core event of vitiligo. In addition to autophagy and ferroptosis, pyrosis, necrosis, anoikis, phahoptosis, and other nonapoptotic programmed cell death modes have been reported recently, which may participate in the pathogenesis of vitiligo. Actually, the characteristics of nonapoptotic programmed cell death have been reported more closely related to vitiligo than apoptosis as previously thought. Studies have shown that NLRP1 (Pyrin Domain containing protein 1) and IL-1*β* (Interleukin-1*β*), important markers of pyrosis, are strongly positive in the skin surrounding lesions of patients with active vitiligo. These results suggest that pyrosis may be involved in the pathogenesis of vitiligo [73]. In addition, the activation of receptor-interacting protein 1 (RIPK1) and RIPK3 are characteristic changes of necrosis. It has been reported that human melanocytes express RIPK3 at a high level, and CD95L-mediated phosphorylation of protein kinase-like domains can induce necrosis of melanocytes [74]. Intrinsic abnormalities and environmental stress probably induce death of melanocytes at the very beginning, which activates innate immune cells through the release of DAMPs. Innate immune cells might then present antigens and trigger adaptive immunity, accelerating the progress of vitiligo. Thus, the destruction of melanocytes which results in skin depigmentation in vitiligo is considered to be multifactorial.

## 4. The Interplay between Oxidative Stress and Cell Death in Vitiligo

Recent investigations have depicted that oxidative stress significantly influences the crosstalk of apoptosis, autophagy, and ferroptosis, and modulates mitochondrial function. The interplay of these cell death modes is very complex. It is well known that at least two sorts of crosstalk of cell death types are affected by antioxidants: crosstalk between autophagy and apoptosis as well as crosstalk between autophagy and ferroptosis. Since apoptosis has been adequately discussed in quite a number of articles, here, we mainly describe the relationship between oxidative stress and autophagy and ferroptosis.

### 4.1. Crosstalk between Autophagy and Oxidative Stress

There have been debates on the crosstalk between redox-dependent signaling pathways and autophagy. Over the past years, ample experimental evidence has indicated that alterations in cellular redox state could be a trigger or regulator of autophagy, and in contrast, autophagy could be a mechanism for regulating mitochondrial redox metabolism [75].

**Figure 1:**
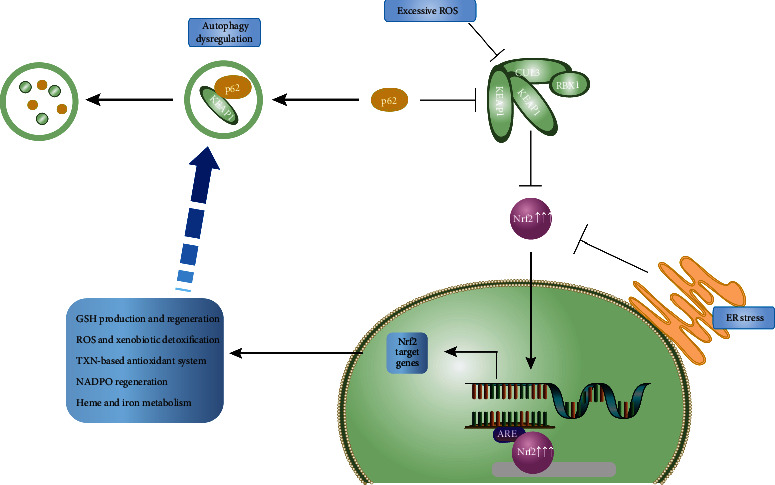
Crosstalk between autophagy and Nrf2 pathway under oxidative stress. When Nrf2 pathway is activated following exposure to excess ROS, or autophagy dysregulation, Nrf2 translocates to the nucleus and binds the ARE to activate the transcription of its target genes. Examples of the general processes regulated by Nrf2 target pathways are indicated. ROS: reactive oxygen species; ER: endoplasmic reticulum.

More and more researches have focused on the possible association between autophagy and melanosome biogenesis, formation, and destruction, which indicated that impairment of autophagy has implications for melanocyte dysfunction and development of skin pigmentation disorders [76, 77]. Recently, HSF1 was confirmed to ameliorate oxidative stress-induced melanocyte death through the activation of autophagy by upregulating ATG5 and ATG12, the critical components for autophagosome formation [78]. Autophagy is required for suppressing the activation of the Nrf2-dependent stress response, maintaining the proliferative potential, and preventing premature senescence of melanocytes. Selective autophagy substrate p62 activates the stress-responsive transcription factor Nrf2 through the inactivation of Keap1. P62 interacts with the Nrf2 binding site on Keap1. Thus, an overproduction of p62 or an autophagy deficiency competes with the interaction between Nrf2 and Keap1, resulting in stabilizing Nrf2 and activating transcription of Nrf2 target genes, including a family of antioxidant proteins [79, 80]. Also, it was demonstrated that dysregulated autophagy owing to the impairment of the Nrf2-p62 pathway increased the sensitivity of vitiligo melanocytes to oxidative stress, thus, promoted the development of vitiligo [21]. We previously uncovered that in both humans and murine melanocytes, deficiency of ATG7-dependent autophagy can lead to premature growth arrest, ROS accumulation, and overactivation of the Nrf2 signaling pathway [46, 81]. Subsequently, we linked the biological functions and the redox homeostasis of melanocytes to the autophagy process, proving that overexpression of autophagy protects melanocytes from oxidative stress-induced apoptosis. Atg7-dependent autophagy is responsible for regulating the Nrf2-ARE signaling pathway of melanocytes under oxidative stress and is involved in the balance between oxidative stress and the antioxidant defense system. Nrf2 target genes were increasingly expressed, and the activity of antioxidant defense components was significantly increased in autophagy-deficient primary melanocytes (see Figure 1). In addition, autophagy deficiency can promote oxidative stress-induced apoptosis of primary melanocytes, further suggesting an interaction between autophagy and apoptosis.

Therefore, alterations of redox state impact autophagy, while autophagy may in turn regulate the oxidation metabolism of cells. Specifically, under oxidative stress, ROS produced in melanocytes could induce autophagy and activate the Nrf2 antioxidant pathway, remove toxic molecules, and maintain the redox homeostasis of melanocytes, while persistent stimulation of ROS might eventually lead to inactivation of autophagy and excessive activation of Nrf2 antioxidant pathway, breaking the redox homeostasis of melanocytes, resulting in premature senescence, decreased proliferation, and pigment synthesis, which may be involved in the onset of vitiligo.

### 4.2. Crosstalk between Ferroptosis and Oxidative Stress

Numerous studies have confirmed that oxidative stress plays an important role in ferroptosis. ROS in ferroptosis may come from a variety of ways, including iron Fenton reaction and mitochondrial respiration. The decrease in cysteine due to system *X*_*C*_^−^ suppression affects GSH biosynthesis. Subsequently, lipid peroxidation occurs because GPX4 cannot reduce ROS accumulation, which underlies the initiation of ferroptosis by erastin-induced oxidative stress. GPX can use glutathione (GSH) to reduce peroxide. Compared with saturated fatty acids (SFA), polyunsaturated fatty acids (PUFAs) are more susceptible to attack by ROS. As a member of the GPX family, GPX4 prevents the oxidation of membrane lipid components by reducing lipid peroxides and plays a decisive role in the cell's resistance to ferroptosis. GPX4 has been identified as a core regulator and biomarker of ferroptosis. As a substrate of GPX4, GSH depletion leads to inactivation of GPX4, resulting in an oxidation-antioxidant imbalance, leading to accumulation of ROS, activating lipoxygenase (LOX), and ultimately leading to ferroptosis. Since vitiligo melanocytes are more sensitive to oxidative stress due to their defects in antioxidant mechanisms, a close relationship between oxidative stress and ferroptosis may play a role in the pathogenesis of vitiligo.

Nrf2 is not only a key factor of oxidative stress but also a mediator of the pathophysiological process of ferroptosis. Nrf2 can upregulate the expression of functional subunit human cysteine/glutamate transporter (xCT) of system Xc^−^ and glutathione level, enhance glutamate secretion, restore GPX4 activity, and reduce the sensitivity of cells to ferroptosis [82]. The proper activation of the Nrf2 signal pathway also helps melanocytes to ameliorate ROS and lipid peroxide accumulation. The presence of Nrf2/HO-1 usually ensures the timely removal of extra ROS, and lack of HO-1 is thought to bring about poor tolerance for oxidative stress. However, overexpression of HO-1 has been observed to promote oxidation reversely mainly through the excessive formation of Fe^2+^, thus, resulting in ferroptosis [83]. Considering that an increased level of Fe^2+^ also causes ROS accumulation in melanocytes, it is worthy of exploring whether Nrf2/HO-1 exerts bidirectional biological effects on melanocytes.

Oxidative protein folding (OPF), the most common form of posttranslational protein modification in the ER, induces the accumulation of misfolded proteins under excess ROS production, triggering ER stress [84]. Under ER stress, RERK acts as a sensor that activates UPR, whose activation is related to ferroptosis [85]. The System Xc^−^ inhibitors erastin and sorafenib activated the PERK pathway, in which the phosphorylation of eukaryotic translation initiation factor 2*α* (eIF2*α*) and the increase in the activating transcription factor 4 (ATF4) in HT-1080 cells [86]. Therefore, erastin and sorafenib may induce ferroptosis by obstructing System Xc^−^ and concomitantly initiating ER stress. Moreover, whereas we know that excess ROS in the ER is derived from OPF and ETCROS [84], whether and how ROS accumulation, which is a core factor in ferroptosis, participates in ER stress and UPR remain to be explored. This attests to the protective and negative feedback mechanism of the PERKATF4-HSPA5-GPX4 pathway in dihydroartemisinin-induced ferroptosis. In tumor cells, Nrf2 can be stimulated by activated PERK to form a positive feedback loop associated with p62/SQSTM1 to promote ARE expression [87]. Hence, unfolded protein forms misfolded protein via OPF under excess ROS, initiating ER stress. Under ER stress, the activated sensor PERK elicits the UPR, inducing transcriptional effects of Nrf2 and ATF4 to resist ferroptosis. The resistant effects benefit from transcriptional production, including ARE, the positive feedback loop related to p62, and the increasing activity and expression of GPX4.

Nrf2 also regulates key proteins in iron metabolism, such as transferrin and ferritin [88]. The regulation of intracellular iron homeostasis involves multiple pathways, among which iron regulatory protein/iron-responsive element is the classic system. The paradoxical effects of Nrf2 have yet to be elucidated. As Nrf2 also modulates genes related to lipid homeostasis, the Nrf2-lipid peroxidation-ferroptosis axis is currently being gradually but incompletely established in vitiligo [72]. Hence, Nrf2 lies at the intersection of iron homeostasis, lipid homeostasis, and redox homeostasis, which urges us to seek the precise niche of Nrf2 in melanocyte ferroptosis and to explore its potential for vitiligo treatment.

### 4.3. Adjustment of Autophagy to Ferroptosis

The cells that undergo ferroptosis are different from cells damaged by traditional oxidative stress due to abnormal changes in iron metabolism. Under physiological conditions, the degradation of ferritin to release Fe^2+^ does not increase the unstable iron pool (LIP) but helps to maintain iron homeostasis and supply the iron demand of cells [89]. However, when a large amount of Fe^2+^ enters the cytoplasmic LIP without buffer, it will cause iron overload and promote the cell to ferroptosis. Autophagy exerts vital effects on ferroptosis via multiple mechanisms, including (a) ferritinophagy, the NCOA4-dependent degradation of ferritin, accumulates ferrous iron to induce ferroptosis, which is found to be induced by erastin [90]. The content of ferritin in the ferroptosis is reduced, and the level of Fe^2+^ is increased. Blocking autophagy or ferritinophagy can significantly reduce the degradation of ferritin and thus alleviate ferroptosis. RNA binding proteins (such as ELAV-like RNA binding protein (ELAVL1) and ZFP36), ER stress (such as p62), and mitochondrial oxidative stress (such as MtROS) are known to have critical effects on ferritinophagy [91]. (b) Mitophagy, the degradation of impaired mitochondria via the gathering of PINK1 and PRKN as well as the transport of cargo receptors, produces “killing ROS” to advance ferroptosis [92, 93]. (c) CMA degrades its substrate GPX4 through the interaction between Lamp-2a located in lysosomes and the GPX4-HSC70-HSP90 trimer, resulting in ferroptosis [94]. (d) Lipophagy, the RAB7A- and ATG5-dependent degradation of lipid droplets, increases the level of fatty acids [95]. (e) Clockophagy, which is critical for ferroptosis, can selectively degrade the core circadian protein ARNTL through autophagy [96].

Taken together, it has been clearly shown that alteration of autophagy plays a role in vitiligo melanocytes loss. Considering that autophagy constantly serves as the upstream regulator of ferroptosis through a variety of pathways, it is reasonable to speculate that the process of melanocyte loss in vitiligo may further involve ferroptosis. Current research progress has provided innovative strategies for treatment targeting autophagy and ferroptosis in vitiligo. Further research is needed to explore the correlation between autophagy and ferroptosis in the loss of melanocytes in vitiligo, as well as their key markers and pathways. Moreover, manipulation of diverse cell death interactions by mitochondrial oxidative stress-linked antioxidants may serve as a critical melanocyte-protective mechanism, as well.

## 5. Conclusion

**Figure 2:**
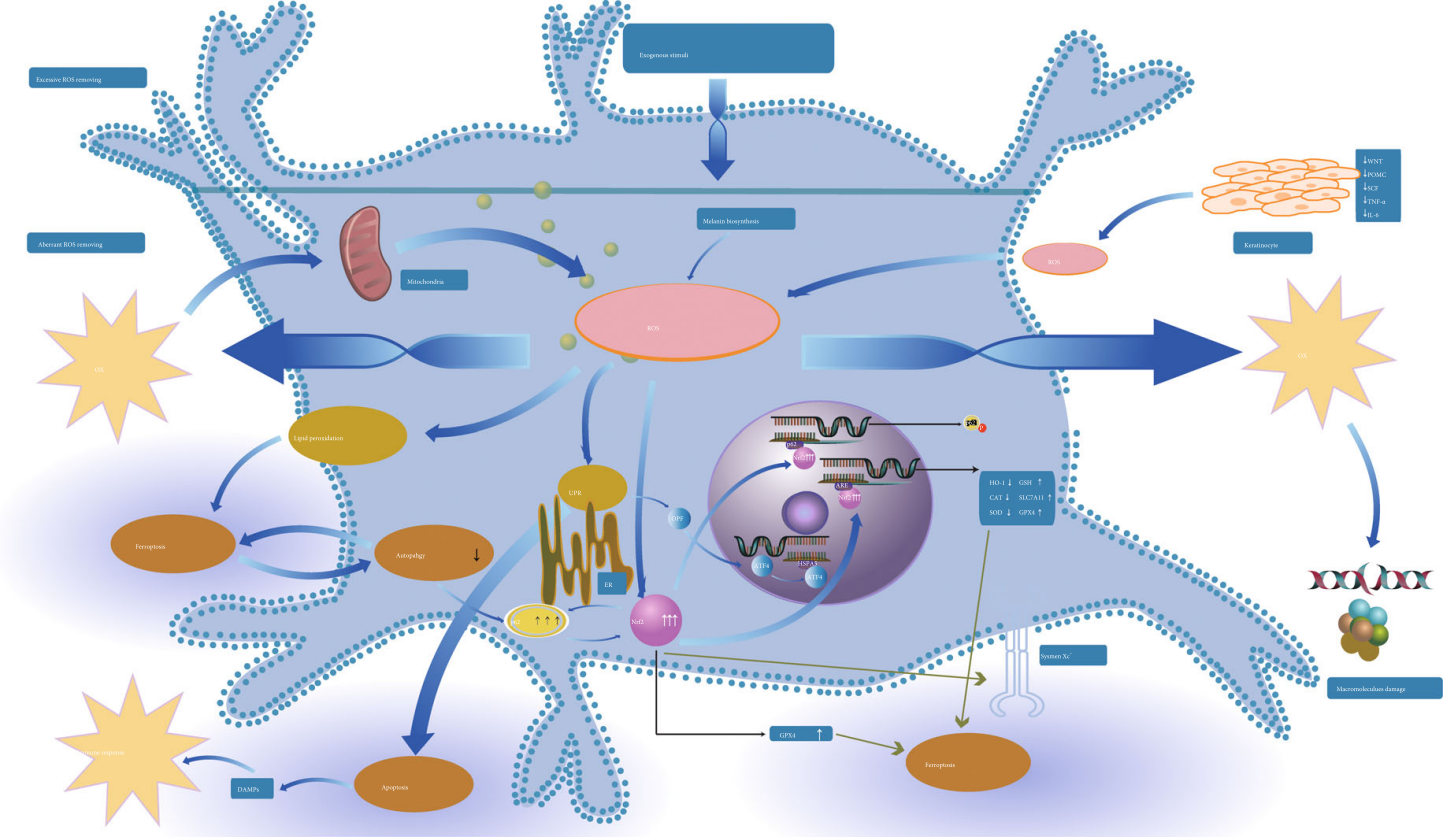
Oxidative stress of melanocytes in vitiligo and its association with multiple cell death modes. Oxidative stress is caused by endogenous and exogenous stimuli that induce melanocytes to produce excessive ROS. Oxidative stress results in various changes of biomolecules, organelles, and keratinocytes, which may ultimately lead to melanocyte apoptosis, autophagy, ferroptosis, and other cell death modes and induce immune responses. Different forms of cell death may be closely related and promote skin depigmentation through their combined effects. ROS: reactive oxygen species; OX: oxidative stress; UPR: unfolded protein action; Nrf2: nuclear factor E2-related factor 2; ER: endoplasmic reticulum; DAMPs: damage-related molecular pattern; GPX4: glutathione peroxidase 4; p62: sequestosome 1/SQSTM1; HO-1: heme oxygenase-1; CAT: catalase; SOD: superoxide dismutase; GSH: glutathione; System Xc^−^: systeine glutamate antiporter; ARE: antioxidant response element.

Oxidative stress plays an important role in initiating the destruction of melanocytes and impairment of redox balance could be one possible mechanism of vitiligo. As the initiating factor of vitiligo, oxidative stress induces various changes in keratinocytes, leading to the death of melanocytes. Although there have been various modes of cell death, and apoptosis has been considered the main mode of melanocyte death in vitiligo, we mainly discuss autophagy and ferroptosis in vitiligo that is closely related to oxidative stress. Since autophagy possesses a protective and restorative function to the damage induced by oxidative stress, deficiency of autophagy might lead to alteration of cell functions, especially on ROS scavenging. Based on the similarity between Atg7 deficiency and vitiligo phenotypes, especially concerning the activation of Nrf2 regulated genes, oxidative stress, and premature senescence, it is very likely that autophagy-deficient melanocytes and vitiligo melanocytes share defective cellular redox regulation, increased membrane lipid oxidation, and premature senescence. Whether ferroptosis occurs and plays a role in the pathogenesis of vitiligo in patients with vitiligo remain to be determined. However, since the oxidative stress mechanism of vitiligo is similar to many diseases where the role of ferroptosis has been well established, we can expect future research on the mechanism of ferroptosis in vitiligo. Last but not least, different forms of cell death may share similarities and be closely connected thus contributing to the outcome through their integrated effects (see Figure 2). Since oxidative stress probably participates in these death modes of melanocytes in vitiligo and triggers a further cascade of reactions leading to the final depigmentation, and it is conceivable to precisely manipulate oxidative stress and to restore redox homeostasis, thus, prevent melanocytes from dying. Further researches delineating the mechanisms of above correlation between oxidative stress and cell death forms will shed light on the mechanisms of melanocyte loss and, more importantly, provide a brand-new direction for us to explore new therapeutic targets of vitiligo.

## Data Availability

No data were used to support this study.
